# Emotional vs. Neutral Face Exploration and Habituation: An Eye-Tracking Study of Preschoolers With Autism Spectrum Disorders

**DOI:** 10.3389/fpsyt.2020.568997

**Published:** 2021-01-13

**Authors:** Aurélie Bochet, Martina Franchini, Nada Kojovic, Bronwyn Glaser, Marie Schaer

**Affiliations:** ^1^Psychiatry Department, Faculty of Medicine, University of Geneva, Geneva, Switzerland; ^2^Fondation Pôle Autisme, Geneva, Switzerland

**Keywords:** emotional faces, habituation, eye-tracking, preschoolers, ASD, face exploration

## Abstract

Diminished orienting to social stimuli, and particularly to faces, is a core feature of autism spectrum disorders (ASDs). Impaired face processing has been linked to atypical attention processes that trigger a cascade of pathological development contributing to impaired social communication. The aim of the present study is to explore the processing of emotional and neutral faces using an eye-tracking paradigm (the emotional faces task) with a group of 24 children with ASD aged 6 and under and a group of 22 age-matched typically developing (TD) children. We also measure habituation to faces in both groups based on the presentation of repeated facial expressions. Specifically, the task consists of 32 pairs of faces, a neutral face and an emotional face from the same identity, shown side by side on the screen. We observe differential exploration of emotional faces in preschoolers with ASD compared with TD. Participants with ASD make fewer fixations to emotional faces than their TD peers, and the duration of their first fixation on emotional faces is equivalent to their first fixation on neutral faces. These results suggest that emotional faces may be less interesting for children with ASD. We also observe a habituation process to neutral faces in both children with ASD and TD, who looked less at neutral faces during the last quarter of the task compared with the first quarter. By contrast, TD children show increased interest in emotional faces throughout the task, looking slightly more at emotional faces during the last quarter of the task than during the first quarter. Children with ASD demonstrate neither habituation nor increased interest in the changing emotional expressions over the course of the task, looking at the stimuli for equivalent time throughout the task. A lack of increased interest in emotional faces may suggest a lack of sensitivity to changes in expression in young children with ASD.

## Introduction

Autism spectrum disorders (ASDs) are a group of neurodevelopmental disorders characterized by persistent deficits in social communication and interactions, as well as restricted and repetitive patterns of behavior, interests, or activities. Symptoms of autism are present early in development and are known to cause variable functional impairment (1). Diminished orienting to faces and reduced eye contact are two striking behavioral hallmarks of autism. Faces are highly salient but complex social stimuli that are essential to social interactions (2). A recent review and meta-analysis of eye-tracking studies confirmed overall reduced attention to social stimuli and impaired face orienting in individuals with ASD compared with typically developing (TD) controls (3). Some authors have suggested this lack of social orientation as a potential trigger for the developmental cascade that contributes to autistic symptoms (4).

Eye tracking is a non-invasive tool, facilitating the detailed characterization of looking behavior (5–7). It is especially useful for screening and detecting early visual exploration abnormalities. Several authors have suggested that eye-tracking measurements may be useful for the diagnosis of ASD (6) or as predictors of outcome (8). For example, Frazier et al. (6) created an eye-tracking-based autism risk index including time spent on different biological and social stimuli. They found that the autism risk index was strongly associated with the severity of autistic symptoms among children between 3 and 8 years old, according to Autism Diagnostic Observation Schedule—Second Edition (ADOS-2) severity scores. To study predictors of outcome, Franchini et al. (8) reproduced a visual preference paradigm by presenting biological and geometric motion side by side during eye tracking. The authors showed that children with ASD who preferred biological motion at the age of 3 showed drastic reductions in the severity of their autistic symptoms 1 year later than did preschoolers who preferred geometric motion (8).

Eye-tracking technology also is an extremely useful tool for understanding autistic symptoms and face exploration behavior in children with ASD. An eye-tracking study detected atypical face exploration for preschoolers with ASD, noting that they looked less at the inner facial features of neutral faces and more at the external facial features than did TD children (9). Two other studies suggested that children with ASD looked significantly less at the eyes and more at the mouth than TD children when viewing social scenes (10, 11). Given the potential for early emergence of autistic symptoms, a few studies have explored face exploration in 2- to 6-month-old infants at risk for ASD, with inconsistent results (12–14). Jones and Klin (13) showed that infants later diagnosed with ASD demonstrate decreased fixations on the eyes within the first 2–6 months of life (13). However, a longitudinal study by Young et al. (14) showed that none of the infants with lower rates of gazing at the eye region at age 6 months had any signs of autism at 18 months (14). Moreover, context appears to modify how toddlers with ASD process faces (15). The authors showed that children with ASD spent less time looking at whole faces and lip movements than did their peers solely in the context of a direct interaction (15).

The mechanisms underlying impaired face processing in ASD are not yet fully understood. Face exploration in ASD appears to change to some degree based on the emotion expressed on the face and whether the face is static or dynamic [for a review, see (16)]. A recent study among adults with ASD linked impaired face exploration with specific deficits in emotion exploration (17), and emotion exploration has been related to difficulties in the recognition of different emotions (18). A few studies have explored emotion recognition in preschoolers with ASD, using either facial or vocal stimuli, with mixed results. Some studies do not point to significantly impaired emotion recognition in individuals with ASD (19), whereas others identify impaired emotion recognition overall or for specific emotions (18), depending on the type of stimuli (18) and characteristics related to the participants (20).

A growing body of work has focused on attentional processes, such as habituation or visual disengagement, and their contributions to socio-communicative impairments in autism (21–24). Hypotheses for explaining both decreased social interest and the developmental cascade of socio-communicative impairments have emerged from recent work exploring attention deficits in children with ASD (21–24). Studies demonstrate impaired visual disengagement, which can result in sticky attention in the absence of habituation to stimuli (25–28). Habituation is defined as the diminishing of a response to a repeated stimulus (29) and is referred to as repetition suppression in neuroimaging studies (30). Some authors have suggested that impaired habituation to sensory stimuli in children with ASD may result in stimulus overload (21, 22). Overwhelmed by their surroundings, children with ASD may have trouble evaluating both the salience and relevance of stimuli, including social cues (21, 22). As a result, social stimuli can be labeled “unpleasant” and, consequently, avoided, contributing to socio-developmental impairment in children with ASD (21, 22). These results are supported by neuroimaging studies. Ewbank et al. (31) showed repetition suppression, with reduced blood oxygenation level-dependent (BOLD) response, and changes in connectivity between the occipital face area and the fusiform face area in neurotypical adults while looking at repeated identical faces (31). The same authors later observed decreased repetition suppression in the fusiform face area in adults with ASD looking at repeated faces (32). Other neuroimaging studies also point to reduced habituation involving the amygdala in individuals with ASD, related to more severe social impairment (33, 34).

During eye tracking, habituation is typically defined by a progressive decrease in looking time at a stimulus when it is repeated (35). However, despite its relevance, surprisingly few eye-tracking studies have investigated differential habituation in children with ASD (35) or habituation to faces (36). A recent eye-tracking study compared habituation responses with sensitivity to novelty in ASD, Williams syndrome, and TD preschool children based on repeated vs. novel geometric stimuli presented side by side (35). The TD and Williams syndrome groups showed reduced attention to the repeating stimuli and increased attention to the novel stimuli over time, while the ASD group showed a similar decrease in attention to both stimuli. Correspondingly, another study showed slow visual habituation to faces in children with severe autism (36). The authors defined habituation when the average of two consecutive visual fixations dropped below 50% of the average of the same child's longest two fixations. Children with ASD needed more trials than controls to habituate to faces compared with houses. To the best of our knowledge, previous studies have never compared habituation with neutral vs. emotional faces in children with ASD. Through the repetition of 32 pairs of neutral and emotional faces, we were able to observe changes in participants' attention to faces and whether habituation to neutral and emotional faces is different between children with ASD and their TD peers.

The aim of this eye-tracking study is two-fold. First, we describe the exploration patterns of emotional and neutral static faces stimuli in children younger than 6 years with ASD and their age-matched TD peers. Namely, we look at the time spent on the faces and inner facial features (eyes and mouth), as well as the number of fixations children made to these areas of interest (see section Emotional and Neutral Face Exploration paragraph for details). The number of fixations, together with fixation duration, allows us to explore whether the child fixates the same part of a stimulus for a prolonged time or whether he/she explores it by making several fixations within the stimulus. This is especially interesting since a “sticky” pattern of exploration is described as one of the early signs of ASD in children (37).

Commensurate with results from previous work (3), we hypothesize that children with ASD will show decreased orienting to faces, especially to emotional vs. neutral faces, than will TD children. We also hypothesize that children with ASD will present a differential exploration pattern, depending on whether a stimulus is emotional or neutral, as compared with TD participants. Specifically, we expect that children with ASD will spend less time looking at the eyes and more time on the mouth than TD children, with a decreased number of fixations in each area of interest (AOI). Second, we will measure habituation during face exploration. In the current study, we define habituation as a significant decrease in the time spent on faces during the last quarter of the task compared with the time spent on faces during the first quarter of the task (see section Habituation paragraph for details). According to recent theories and previous results (35, 36), we hypothesize that children with ASD will show reduced habituation to faces, both emotional and neutral, spending a similar amount of time looking at faces throughout the task.

## Materials and Methods

### Participants

The present study included 24 participants with ASD (2F), 1.88–5.63 years old (3.92 ± 1.18), and 22 TD children (6F), 1.77–5.84 years old (4.12 ± 1.08) (Table 1). Both groups did not differ by age (*p* = 0.547) or gender (*p* = 0.128).

**Table 1:** ASD (Autism Spectrum Disorder) and TD (Typically Developing) groups characteristics.

	**ASD group**	**TD group**	***p*-value** **[*t*_**(df)**_]**
*N* (Gender)	24 (Males = 22/Females = 2)	22 (Males = 16/Females = 6)	0.128 (Fisher's exact-test)
Age Mean ± SD (Range)	3.92 ± 1.18 years old (1.88–5.63 years old)	4.12 ± 1.08 years old (1.77–5.84 years old)	0.547 [*t*_(44)_ = 0.61]

Participants with ASD were recruited through local ASD-specialized therapeutic centers and parent associations. For participants with ASD, the clinical diagnosis was confirmed using either the ADOS-Generic (ADOS-G) (38) or the ADOS-2 (Second Edition) (39) (Table 2). The ADOS consists of a series of structured and semi-structured tasks involving social interactions between an examiner and a child. It serves as a standardized tool for diagnosing ASD and quantifies observations in three domains of behavior: communication, social interaction, and repetitive/restricted behaviors or interests. The version (module) of the ADOS is selected according to a child's age and language level. Therefore, the ADOS module differed between participants. Accordingly, the ADOS Calibrated Severity Score served as a standardized measure of symptom severity across modules (40). Calibrated Severity Scores also were obtained for the Social Affect (SA-SS) and the Restricted Interests and Repetitive Behaviors domains (RRB-SS). The 22 participants with typical development were recruited through announcements in the Geneva community. They also were assessed using the ADOS-G or ADOS-2 to ensure the absence of ASD symptoms prior to their inclusion in the TD cohort. All TD children had a Total Severity Score of 1 (the minimum), except for one child who had a score of 2. Children also were excluded from the TD group if they presented any neurological/psychiatric problems or learning disabilities according to a parent interview and questionnaire, or if they had a sibling or first-degree relative diagnosed with ASD. The study was approved by the Local Research Committee, the Commission Centrale d'Ethique de Recherche (CCER) in Geneva, Switzerland, and written informed consent was obtained from a parent of all the participants prior to inclusion in the study. All participants are part of the Geneva Autism Cohort, a cohort of preschoolers followed up longitudinally (8, 41).

**Table 2:** ADOS calibrated severity scores and best estimate intellectual quotient for ASD (autism spectrum disorder) and TD (typically developing) groups.

	**ASD group**	**TD group**	***p*-value** **[*t*_**(df)**_]**
Total ADOS severity score Mean ± SD	7.21 ± 1.84	1.05 ± 0.21	<0.001 [*t*_(44)_ = 15.59]
ADOS social affect severity score Mean ± SD	6.08 ± 1.93	1.09 ± 0.42	<0.001 [*t*_(44)_ = 11.85]
ADOS restricted interests and repetitive behaviors severity score Mean ± SD	8.92 ± 1.38	2.09 ± 1.82	<0.001 [*t*_(44)_ = 14.39]
Best estimate intellectual quotient Mean ± SD	81.51 ± 26.00	122.90 ± 17.43	<0.001 [*t*_(44)_ = 6.29]

*ADOS, Autism Diagnostic Observation Schedule*.

A total of 59 pre-schoolers (all under the age of 6) participated in the current study (31 children with ASD and 28 TD children). Five children with ASD were subsequently excluded because they did not sufficiently attend to stimuli presented during eye tracking. To be included in the statistical analysis, participants needed to attend to at least 50% of trials (16 stimuli) for at least 50% of the presentation duration of each stimulus (2.5 s). We further excluded data from three children (two with ASD) due to a failure in the eye-tracking calibration procedure detected during a visual inspection of the recording (see section Procedures and Experimental Measures paragraph for details). Consequently, to keep the ASD and TD groups matched for gender, we had to exclude five TD girls. After a total of 13 children (7 with ASD) were excluded, the final groups comprised 24 participants with ASD and 22 TD children. Children with ASD who were excluded due to failed eye tracking did not differ on severity of autism symptoms as compared with those who were included in the final sample (mean ADOS Total Severity Score: included participants = 7.21 ± 1.84 and excluded participants = 6.29 ± 2.28).

Cognitive functioning was assessed using several standardized tools. With the use of an approach previously described by our team (42) and commensurate with other studies (43), cognitive tools were selected according to age, language level, symptom severity, and participants' abilities to attend to demanding cognitive tasks. We established a Best Estimate Intellectual Quotient (BEIQ), based on either the Wechsler Pre-school and Primary Scale of Intelligence, fourth edition (WPPSI-IV) (44), the Mullen Scales of Early Learning (MSEL) composite score (45), or the Psycho-Educational Profile, third edition (PEP-3) (46). The WPPSI-IV is a standardized intelligence test for young children, aged between 2.5 and 7 years 7 months. It is used to determine the child's intelligence quotient (IQ) and includes five composite domain scores: the Verbal Comprehension Index score, the Visual-Spatial Index score, the Fluid Reasoning Index score, the Working Memory Index score, and the Processing Speed Index score. The MSEL is a standardized assessment used to measure cognitive functioning in children from the ages of 0 to 68 months. The scale includes five subdomains: Visual Reception, Fine and Gross Motor Skills, and Receptive and Expressive Language. The total composite score gives an estimate of overall intelligence. By comparison, the PEP-3 is a standardized tool for assessing developmental level in children with developmental disorders, and ASD in particular, between the ages of 2 and 7.5 years. It includes the same five domains as the MSEL but combines non-verbal and verbal intelligence scores into a Verbal/Preverbal Cognition score and includes an additional Imitation Skills Index.

Choice between the different scaled scores was as follows: whenever possible, we used the Full-Scale IQ (FSIQ) score from the WPPSI-IV (ASD *N* = 0, TD *N* = 3). For participants with lower cognitive abilities or if the FSIQ derived from the WPPSI-IV was not quantifiable, we used either the MSEL or the PEP-3, depending on when participants were tested for the study. The MSEL was added to our study protocol in 2015 and was used for participants who were evaluated after that date. Thus, the MSEL was used to calculate the BEIQ for 19 participants with ASD and 14 TD participants. The Verbal/Preverbal Cognition scale of the PEP-3 was used to calculate the BEIQ for five participants with ASD and five TD participants. Ten of 24 (42%) participants with ASD obtained a BEIQ score below 70 (range 49.0–68.0). The ASD group had a significantly lower mean BEIQ than the TD group (*p* < 0.001; Table 2).

### Procedure and Experimental Measures

Participants were seated on a chair or on their parent's laps, ~60 cm from the screen. Gaze data were acquired and analyzed using TX300 Tobii eye-tracker software with a 300-Hz sampling rate (https://www.tobiipro.com). The stimuli were presented on a 1,200 × 1,920 pixel video monitor and subtended at a minimal horizontal visual angle of 7.82° and a minimal vertical visual angle of 2.86° for the smallest AOI. All participants were administered a five-point calibration procedure to verify eye motion and gaze presence before beginning the task. Calibration was repeated until the eye tracker correctly detected child's gaze.

The emotional faces task comprised a sequence of 32 stimuli, each composed of two adult faces presented side by side on the screen for a duration of 5 s. Each stimulus included a neutral face and an emotional face taken from the same person/identity. Thus, the task included a total of four emotion expressions (anger, fear, happiness, and sadness), with eight different identities expressing each emotion. Emotions were mixed throughout the task. The face stimuli were all part of the Radboud Faces Database (47), and all emotions were expressed at the same intensity according to the database. Between each stimulus, a rotating wheel was displayed in the center of the screen for 1 s to recenter participants' visual attention. Two additional seconds of the rotating wheel to center participants' attention was required (1 s before the beginning of the task and 1 s at the end of the task). Total task duration was 3 min 13 s. Children did not receive any viewing instruction prior to the task. All participants viewed the sequence of face stimuli in the same order.

### Data Analysis

**Figure 1:**
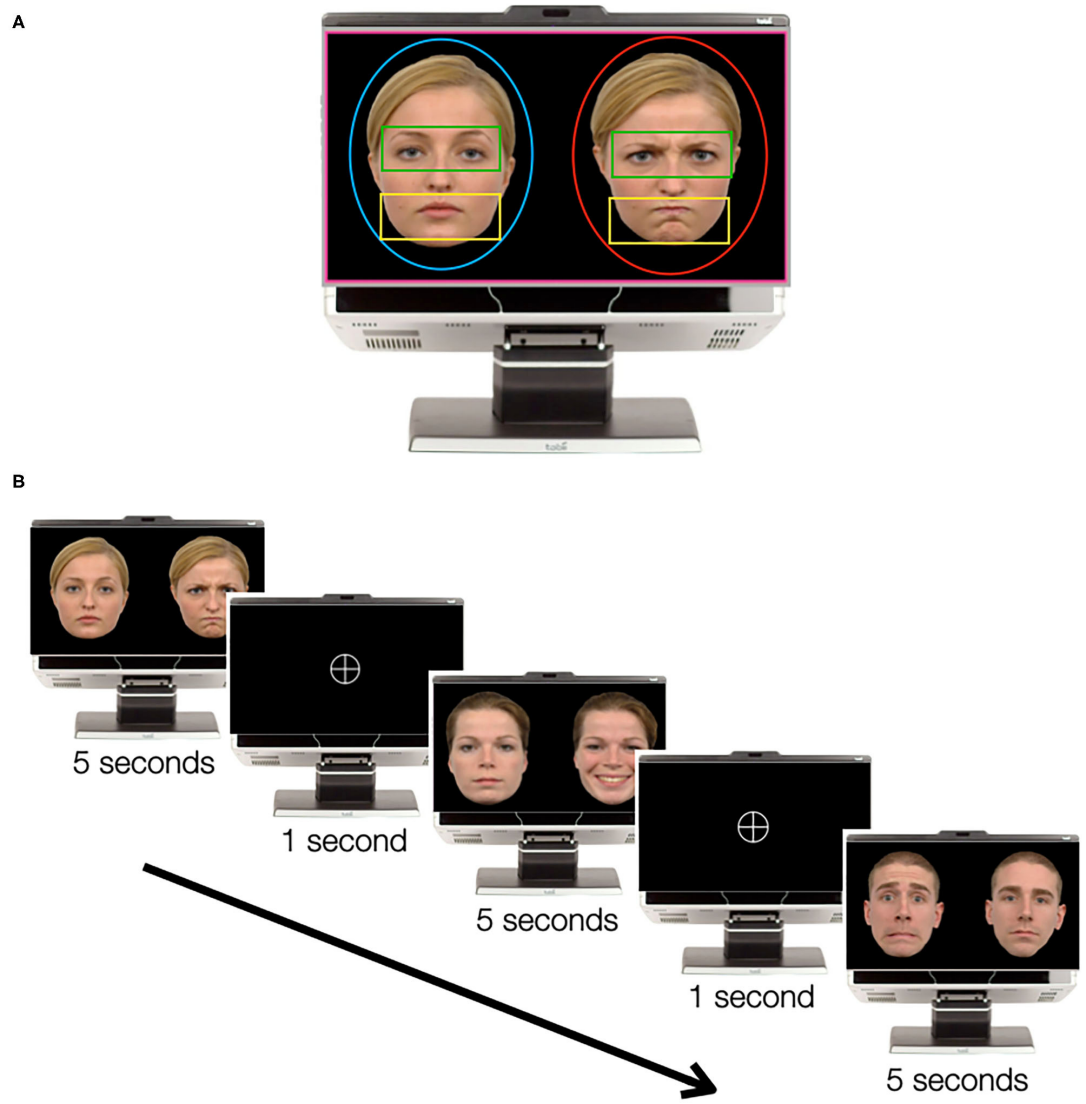
The emotional faces task. **(A)** Example of one pair of faces with area of interest (AOI). AOI representation: screen in pink, neutral face in blue, emotional face in red, eyes in green, and mouth in yellow. The pair of faces was extracted from the Radboud Faces Database (47). **(B)** Example of the order of the stimuli, with three pairs of faces and two turning wheels between the faces.

To quantify the time spent looking at key regions of the faces, we drew an oval-shaped face AOI on each of the side-by-side faces (see Figure 1) and added rectangular-shaped AOI for the eye and mouth regions. We also drew an AOI containing the whole screen to quantify screen attendance (see the following paragraph). The size of the AOI containing the screen and the faces was equivalent for all stimuli. AOI containing the eyes and mouths was adjusted according to the physiognomy of each identity and thus equals for the two faces in each stimulus.

Data analysis was performed using IBM SPSS Statistics for Macintosh, Version 24.0 (IBM Corp., Armonk, NY), and graphs were plotted using GraphPad Prism for Macintosh, Version 8 (GraphPad Software, La Jolla, CA, USA; www.graphpad.com). Normality of distribution was tested with D'Agostino–Pearson test for all the variables. Screen attendance was measured using total time spent on the screen and the number of visits on the screen, allowing us to quantify the amount of time that children looked at the screen after looking away. We used an unpaired *t*-test to compare screen attendance between groups.

Fixation time spent on each group of AOI (all faces, emotional faces, neutral faces, eyes, and mouth) was calculated for the ASD and TD groups. Fixations were defined according to the Tobii IV-T Fixation filter (48), using a velocity threshold of 30° within a velocity window length of 20 ms. Adjacent fixations were merged. The maximum time between all fixations was 75 ms, and the maximum angle between all fixations was 0.5°. Despite the fact that screen attendance was comparable in both groups, we still controlled for overall time spent on the screen during stimulus presentation by calculating the percentage of time children fixated on each AOI group relative to overall time spent on the screen.

#### Emotional and Neutral Face Exploration

A one-way multivariate analysis of variance (MANOVA) was run to determine the effect of diagnosis on fixation duration on emotional and neutral faces. We then compared the overall number of fixations within and between ASD and TD groups, using paired and unpaired *t*-tests, respectively. We used the same approach for duration of first fixations on the neutral face and the emotional face of each stimulus.

To test for differences in the exploration of emotional and neutral faces, we calculated fixation time on the eyes and the mouth of emotional and neutral faces in both groups. Effect of diagnosis on fixation duration by region (eyes or mouth) was then tested using a one-way MANOVA. To end, we tested the interaction between fixation duration for the four emotional expressions and diagnosis, using a two-way ANOVA. Then we performed *post-hoc* analysis using Tukey's multiple comparisons test for fixation duration on each emotion between the ASD and TD groups.

We used Pearson correlations (data were normally distributed) to detect potential relationships between ADOS Total Severity Score and our eye-tracking measures (fixation duration on the screen and on emotional and neutral faces, first fixation duration on emotional and neutral faces, and number of fixations on emotional and neutral faces).

#### Habituation

In the context of the present study, habituation was defined as the difference in time spent on the first quarter of the task (first eight stimuli) compared with the last quarter (last eight stimuli). We calculated fixation duration on each stimulus (including both the emotional and neutral faces), as well as on each emotional/neutral face. The sums were adjusted according to overall time spent on the screen during the first quarter and the last quarter, respectively. We compared the differences in both groups, using paired *t*-tests, and we applied a Bonferroni correction for multiple comparison.

## Results

The difference in total time spent on the screen between the ASD (*M* = 124.95 s, SD = 16.87) and TD groups (*M* = 132.02 s, SD = 13.26) was not statistically significant [*t*_(44)_ = 1.57, *p* = 0.124], nor was the difference in the number of visits to the screen between the ASD (*M* = 35.58, SD = 5.22) and TD groups (*M* = 36.27, SD = 4.81) [*t*_(44)_ = 0.46, *p* = 0.645].

### Emotional and Neutral Face Exploration

Overall, participants with ASD spent an equivalent amount of time looking at faces (*M* = 90.25%, SD = 4.03) as compared with TD participants (*M* = 91.65%, SD = 2.59) [*t*_(44)_ = 1.34, *p* = 0.170].

**Figure 2:**
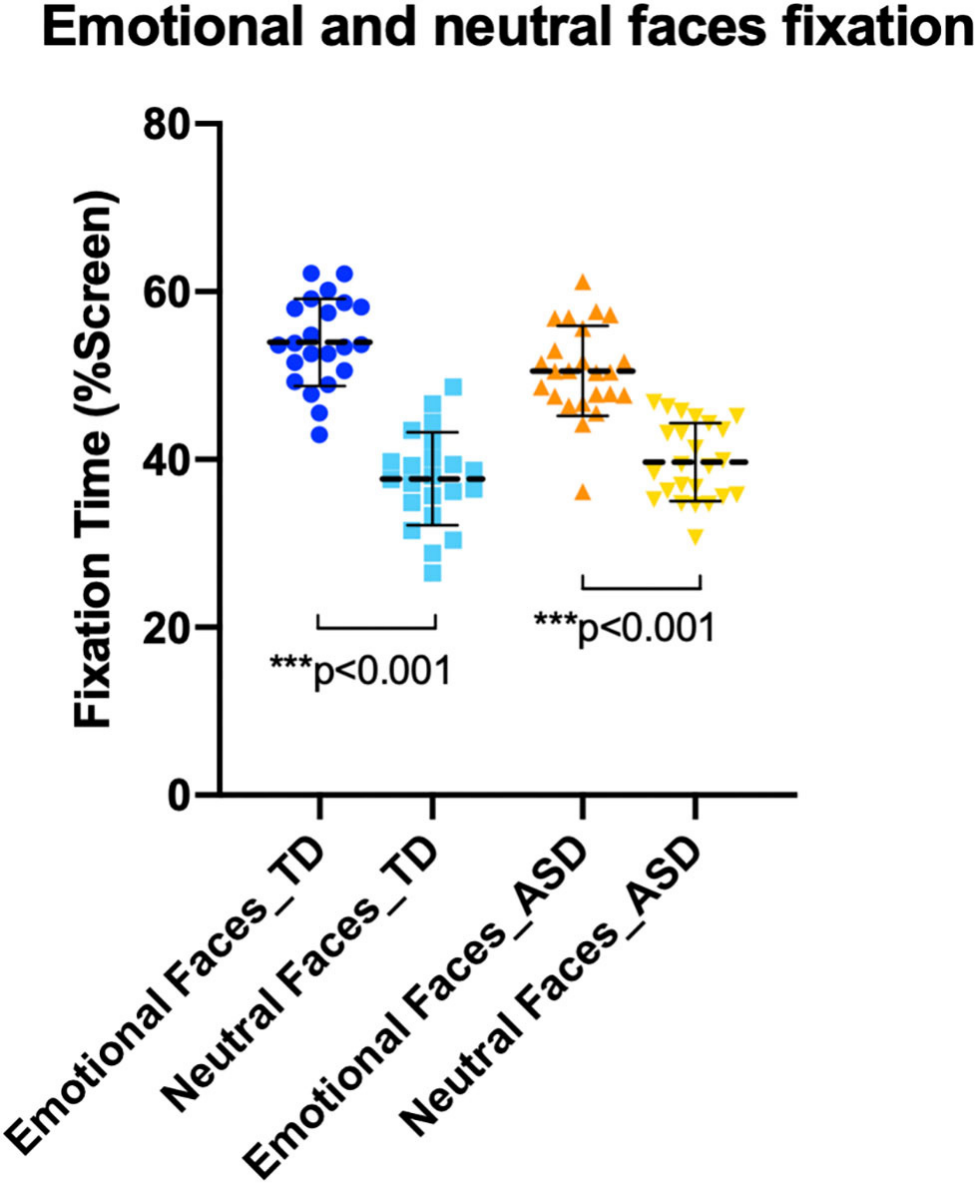
Fixations to emotional and neutral faces in autism spectrum disorder (ASD) and typically developing (TD) group. TD group (on the left) and ASD group (on the right) both looked significantly less at neutral faces than emotional faces.

A one-way MANOVA tested the effect of diagnosis on the exploration of emotional and neutral faces [fixation duration on emotional faces ASD group (*M* = 50.56%, SD = 5.37) and TD group (*M* = 53.96%, SD = 5.20); fixation duration on neutral faces ASD group (*M* = 39.69%, SD = 4.63) and TD group (*M* = 37.69%, SD = 5.55)] (Figure 2). There was a tendency toward a main effect of diagnosis on the combined dependent variables [*F*_(2, 43)_ = 2.501, *p* = 0.094; Wilks' lambda = 0.896; partial η^2^ = 0.104]. Follow-up univariate ANOVA showed that the two groups were statistically different in the total time spent exploring emotional faces [*F*_(1)_ = 4.749, *p* = 0.035; partial η^2^ = 0.097], but not neutral faces [*F*_(1, 44)_ = 1.769, *p* = 0.190; partial η^2^ = 0.039].

**Figure 3:**
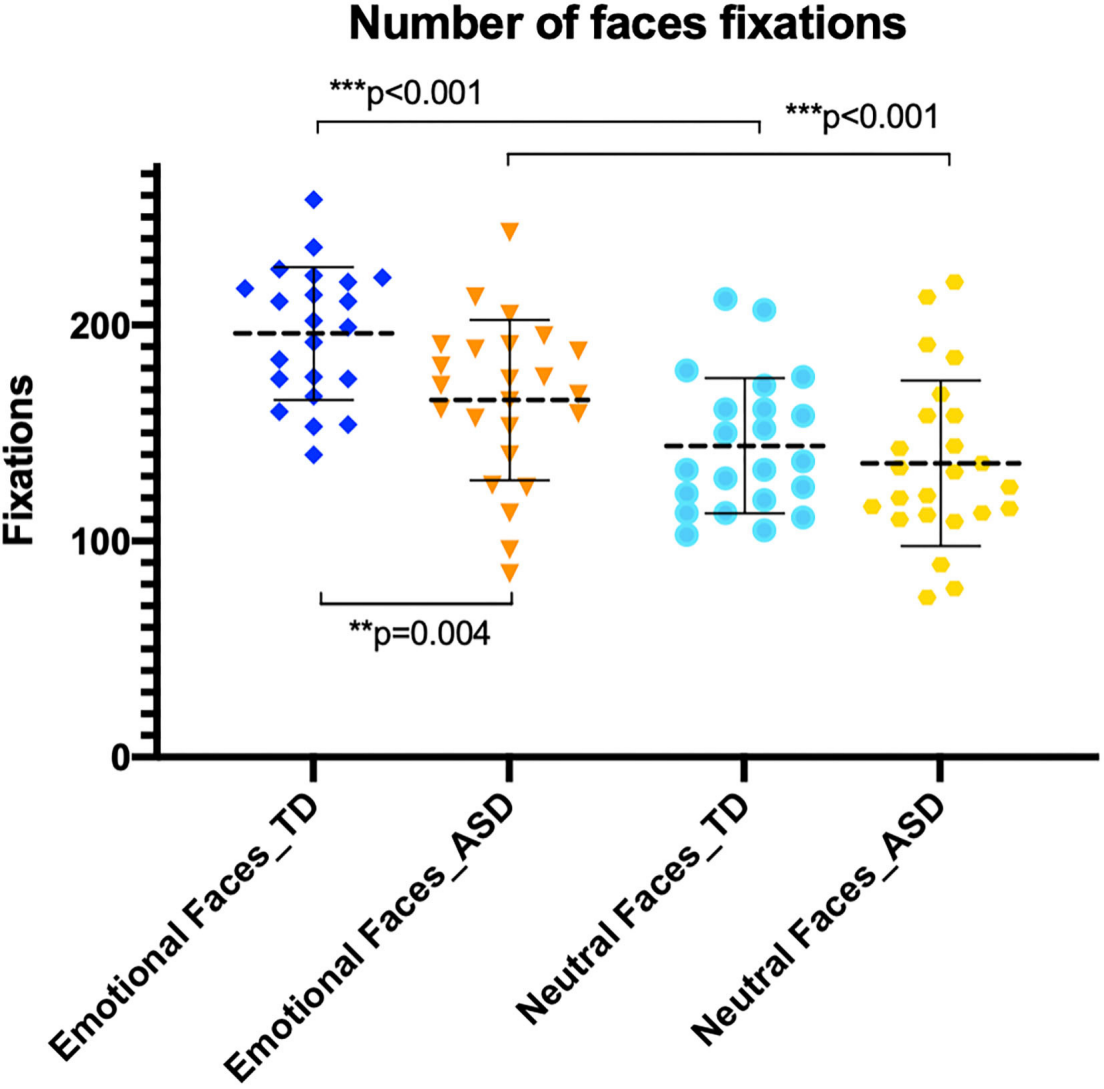
Number of fixations on emotional and neutral faces. Autism spectrum disorder (ASD) group (orange) made less fixations to emotional faces than typically developing (TD) group (dark blue).

Both groups made more fixations to emotional faces than to neutral faces (Figure 3). Participants with ASD made significantly more fixations to emotional faces (*M* = 165.30, SD = 37.15) than neutral faces (*M* = 136.00, SD = 38.39) [*t*_(23)_ = 4.99, *p* < 0.001]. The TD group also fixated more on emotional faces (*M* = 196.10, SD = 30.79) than neutral faces (*M* = 144.10, SD = 31.40) [*t*_(21)_ = 7.11, *p* < 0.001]. However, when we compared both groups, the ASD group made significantly less fixations to emotional faces than the TD group [*t*_(44)_ = 3.05, *p* = 0.004]. No between-group difference was detected in the number of fixations to neutral faces [*t*_(df)_ = 0.78, *p* = 0.438].

**Figure 4:**
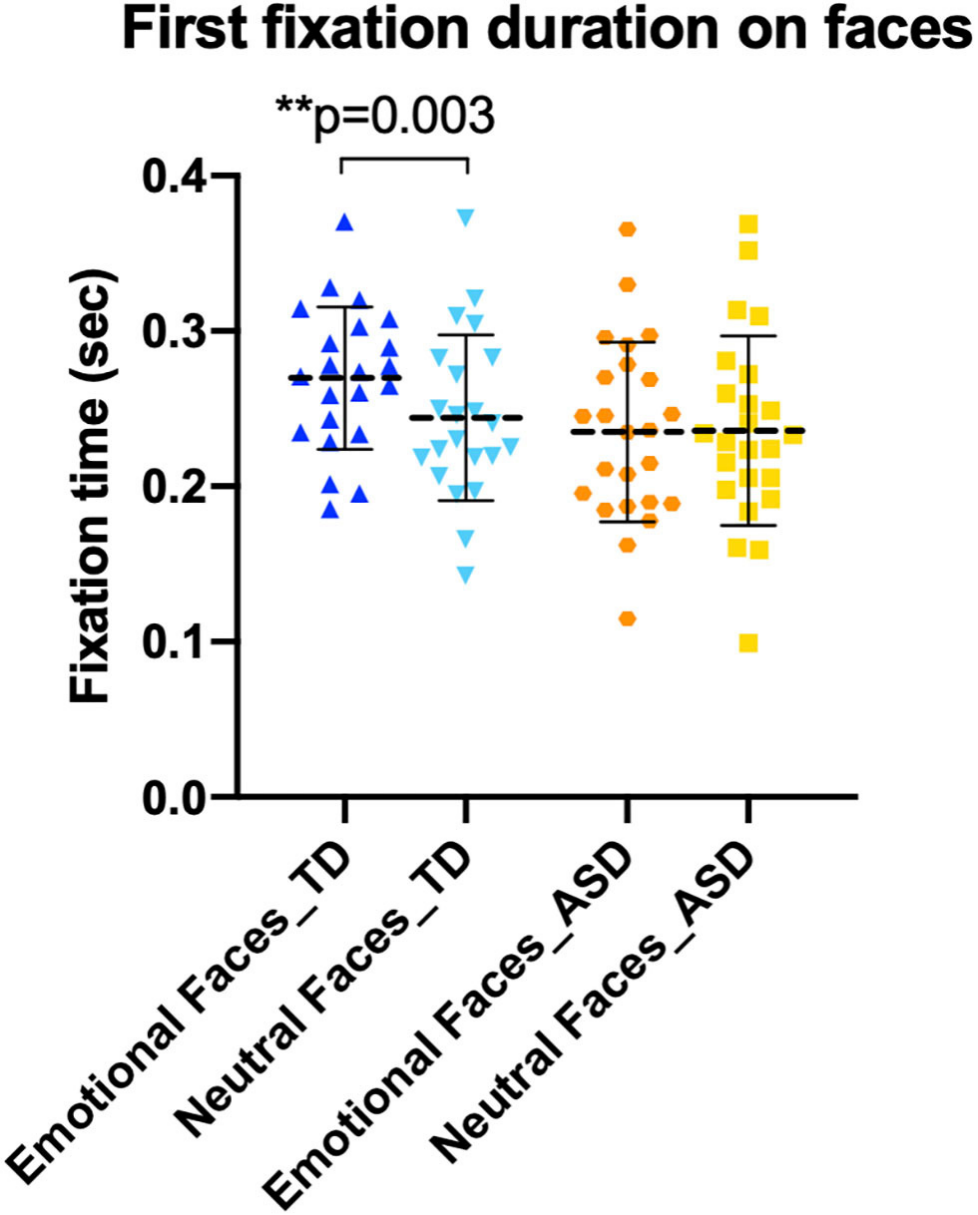
First fixations to emotional and neutral faces. Typically developing (TD) children showed longer first fixation on emotional faces (dark blue) than on neutral faces (light blue).

We did not observe a significant difference [*t*_(23)_ = 0.10, *p* = 0.924] in the first fixation duration between emotional (*M* = 0.24 s, SD = 0.06) and neutral faces (*M* = 0.24 s, SD = 0.06) in the ASD group (Figure 4). However, there was a significant difference [*t*_(21)_ = 3.44, *p* = 0.003] in the duration of first fixations between emotional (*M* = 0.27 s, SD = 0.05) and neutral faces (*M* = 0.24 s, SD = 0.05) in the TD group.

**Figure 5:**
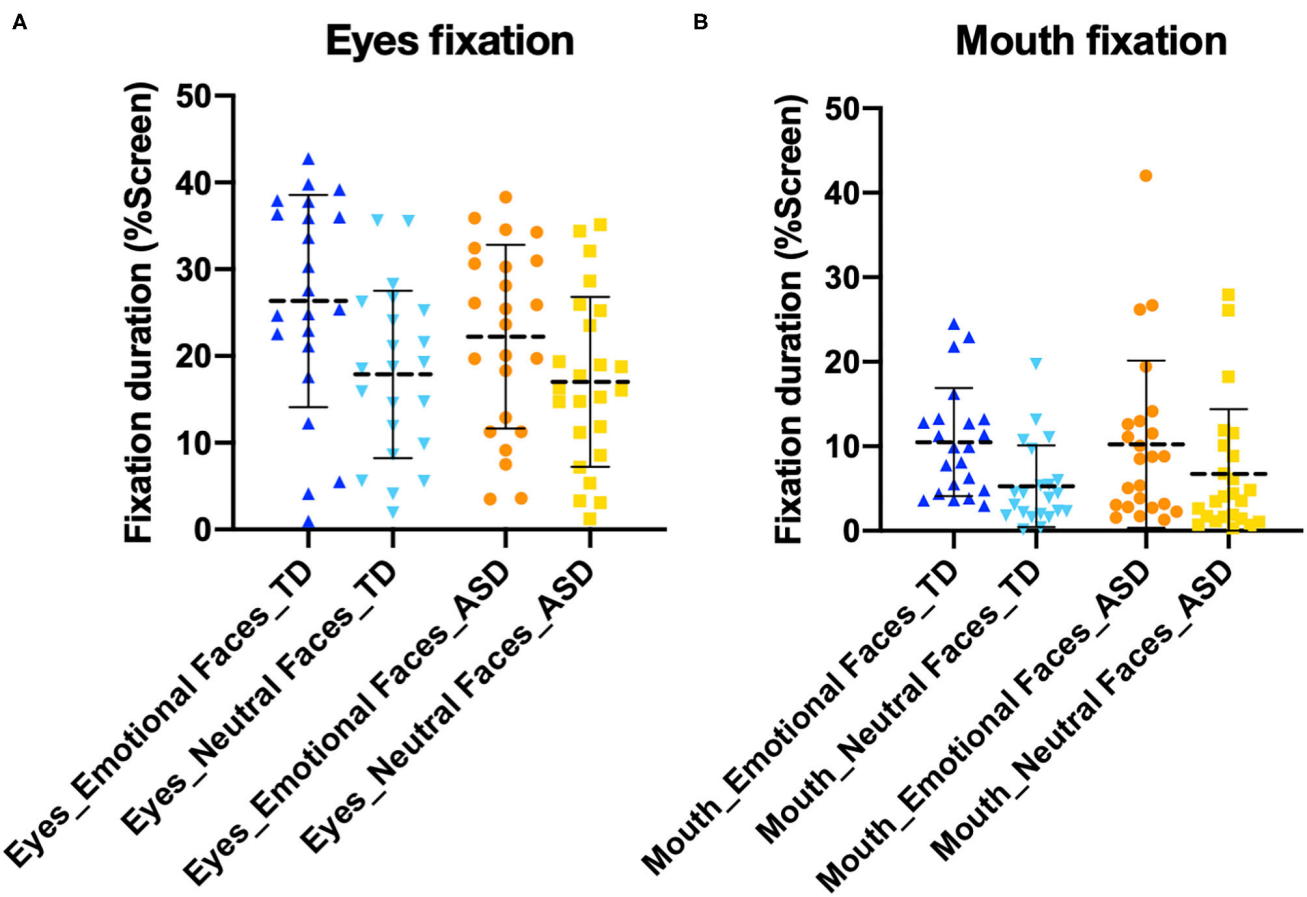
Fixations to **(A)** eyes and **(B)** mouth. There was no difference between the typically developing (TD) and autism spectrum disorder (ASD) groups.

**Figure 6:**
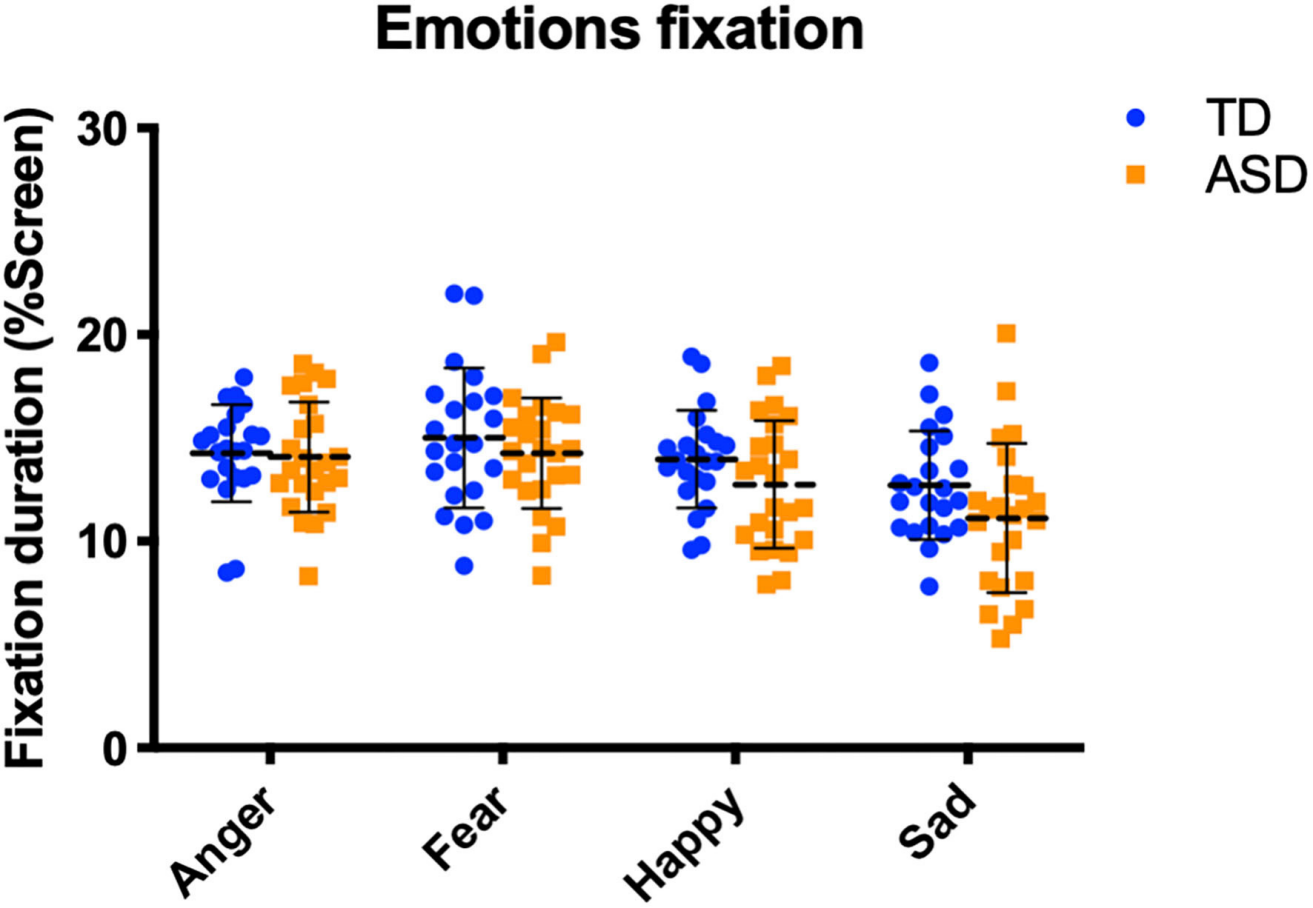
Fixations to the four different emotions. There was no difference between the typically developing (TD) (blue) and autism spectrum disorder (ASD) (orange) groups.

We did not observe an effect of diagnosis on time spent on facial features (Figure 5). In other words, we did not observe a significant difference between time spent on the eyes and mouth, between ASD and TD [*F*_(2, 43)_ = 0.389, *p* = 0.680]. Concerning the four different emotions, there was no significant difference in time spent on emotions between the ASD and TD groups, nor did we observe an interaction between expression (anger, fear, happiness, and sadness) and diagnosis [*F*_(3, 176)_ = 0.52, *p*=0.671] per a two-way ANOVA (Figure 6).

We did not observe any significant correlations between the ADOS Total Severity Score and our eye-tracking measures in the ASD group (all *p* ≥ 0.105).

### Habituation

**Figure 7:**
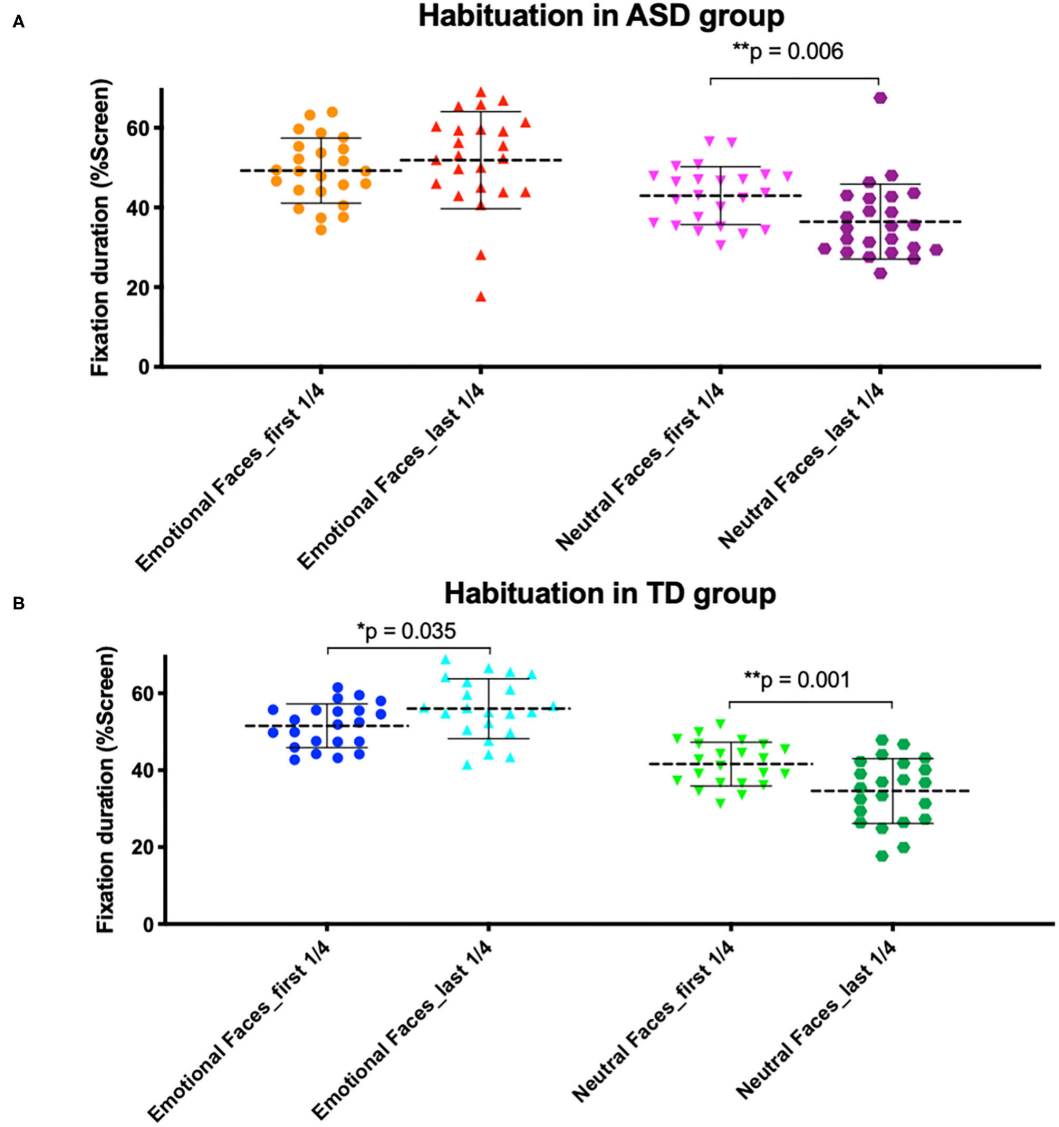
Habituation to emotional and neutral faces in **(A)** autism spectrum disorder (ASD) and **(B)** typically developing (TD) group. **(A)** The ASD group looked less at neutral faces throughout the task. **(B)** The TD group looked less at neutral faces throughout the task but looked more at emotional faces.

We then looked at habituation to faces over the course of the task and found a significant effect of time spent on neutral faces (Figure 7). The ASD group spent less time on neutral faces during the first quarter of the task (*M* = 42.99%, SD = 7.28) than during the last quarter (*M* = 36.45%, SD = 9.42) [*t*_(23)_ = 3.06, *p* = 0.006]. The TD group also spent less time on neutral faces during the first quarter of the task (*M* = 41.61%, SD = 5.69) than the last quarter (*M* = 34.59%, SD = 8.43) [*t*_(21)_ = 3.71, *p* = 0.001].

However, no significant effect of time spent on emotional faces was observed in either group of children after having applied a Bonferroni correction (*p* significant if ≤0.025). The ASD group looked slightly less at emotional faces during the first quarter of the task (*M* = 49.28%, SD = 8.15) than the last quarter (*M* = 51.89%, SD = 12.19); however, the difference was not statically significant [*t*_(23)_ = 1.05, *p* = 0.306]. TD children also looked slightly less at emotional faces during the first quarter of the task (*M* = 51.56%, SD = 5.67) than the last quarter (*M* = 56.02%, SD = 7.79), but the difference was not statically significant [*t*_(21)_ = 2.25, *p* = 0.035]. Both groups looked significantly more at the background of the screen behind the faces during the last quarter of the task than the first quarter (ASD group *p* = 0.008; TD group *p* < 0.001).

## Discussion

Contrary to expectations, our results are not commensurate with previous studies showing altered orienting to faces in preschoolers with ASD compared with TD preschoolers. Further, we did not observe differences in time spent on the eyes or the mouth between subject groups. As suggested by Falck-Ytter and Von Hofsten in a review of literature, the finding that individuals with ASD look less at the eyes and more at the mouth than did TD peers is not consistent between eye-tracking studies of children (49). While it is most frequent among studies based on dynamic stimuli (10, 11), a recent eye-tracking study using dynamic face stimuli and based on large samples of toddlers with ASD, TD, and developmental delay did not show decreased fixations to the eye region in toddlers with autism (50). Moreover, the authors did not observe a correlation between time spent on the eyes and clinical data related to autism severity, language level, or cognitive skills.

In addition, we did not observe a preference pattern between emotional expressions in either participant group. While some studies have observed impairments in emotion recognition and processing in children with ASD (18, 20), to the best of our knowledge, no clear consensus has emerged from the many studies regarding differences in emotion exploration in children with ASD. However, several studies have suggested that “negative” emotions such as fear and anger may be less salient for children with ASD (51–53). For example, a recent eye-tracking study showed that high-functioning children with ASD perceive fear as less intense than their TD peers (52). Moreover, a recent magnetoencephalography study demonstrated differences in electrical activity in children with ASD while looking at happy vs. angry faces, suggesting reduced salience or perhaps an underdeveloped response to anger (51). Finally, an electroencephalography (EEG) study suggested that boys with ASD are less able to detect angry and fearful faces than their TD peers in the absence of group differences on sadness or happiness (53).

Despite a lack of group differences in time spent, we found that our ASD subjects tended to explore emotional faces differently than the TD group. Indeed, ASD participants completed less fixations, as well as shorter first fixations, to emotional faces than TD children, stressing the need for understanding subtle differences in exploration pattern in this population. This may indicate that preschoolers with ASD visually engage less frequently with emotional faces in the absence of a clear difference in the total time spent on the emotional faces, pointing to a somewhat “stickier” pattern of attention. There were no observable intra-group differences in the duration of our ASD group's first fixations on emotional and neutral faces, whereas the duration of first fixations to emotional faces was significantly longer than on neutral faces in the TD group. This could suggest that emotional faces are perceived as less appealing and less salient for children with ASD. This finding is in line with a recent pupillometry study, in which a lack of pupil dilatation was reported in children with ASD while looking at objects, as well as static and dynamic faces (54). The authors attributed this lack of difference to an insensitivity toward event-related pupil dilatation, suggesting an abnormal physiological reaction and reduced arousal to socially salient stimuli in ASD.

Our habituation analysis of looking time over the course of the task revealed that ASD participants' attention to emotional faces does not improve during the task, as expected, despite the novel element of changes in emotional expression between stimuli. In both groups, we found that children attended less to neutral faces over the course of the task, whereas time spent looking at emotional faces did not significantly change between the beginning and end parts of the task though TD children did look slightly more at emotional faces during the last quarter of the task than the first quarter (this difference was not statistically significant). This suggests a somewhat intact habituation response to neutral faces in children with ASD, in the absence of habituation or increased interest in response to emotional faces. This result does not fully support previous eye-tracking studies observing impaired habituation in children with autism (35, 36); however, it does suggest that children with ASD may be impaired in their sensitivity to novelty.

Deficits in habituation and other attention processes, such as disengagement of attention, are now being studied as potential early markers that may contribute to an atypical developmental cascade resulting in socio-communicative impairments (21–24). According to previous studies and reviews, a primary impairment in attention disengagement could lead to a tendency to fixate on social stimuli early in life, decreased habituation preventing the maintenance of a homeostatic brain state leading to either hyperarousal or hypoarousal, and impaired attention shifting and novelty detection (23, 24). Trouble discriminating socially salient and non-salient stimuli could be the natural consequence, which in turn may contribute to impaired face processing (23). Social cues are then marked as unpleasant rather than rewarding, encouraging the avoidance of social stimuli, decreased social interest, and impaired joint attention and executive control (21–24). This also could lead to restricted and repetitive behaviors, as demonstrated in at-risk children with ASD as a strategy for coping with hyperarousal (21).

Further eye-tracking studies will be necessary to understanding the habituation response in young children with ASD, given the clinical heterogeneity in the ASD population (35) and the need to better understand attention processes that may precede decreased social orienting in at-risk ASD toddlers. Such behavioral markers could guide the development of new diagnostic tools and early interventions that target key behaviors. Hence, understanding the subtleties of altered attention processes in children with ASD could help determine their putative role in the core ASD features.

### Limitations

The current study has some limitations. First, the sample size is quite small and, unfortunately, prevented us from separating the ASD group in subgroups based on clinical heterogeneity. The small sample size was due to the relatively long length of the task, and some participants dropped out. The children were excluded from the study because they did not complete enough of the task and did not differ in autism symptom severity from those who were included. However, we cannot guarantee that this did not bias our sample. Our small sample is also related to the fact that we applied very strict study inclusion criteria. Also, we defined habituation differently, by using the difference in time spent on faces during the first quarter of the task compared with the last quarter in order to observe changes over the course of the entire task. Given that the face identities changed between stimuli, the emotional face was not the only novel element, and the neutral face was not identical from trial to trial; however, given our choice of photos, the change in emotional expression was the most striking change.

## Conclusion

In this eye-tracking study, we explored emotional and neutral face processing. Over the course of 32 stimulus repetitions, we observed the presence of the habituation response to social stimuli in a group of children with ASD aged 6 or younger. We found that children with ASD looked differently at emotional faces than TD children and that emotional faces were less interesting to our participants with ASD than predicted. We also observed a habituation process to neutral faces in children with ASD, in the absence of habituation to or increased interest in emotional faces throughout the task, suggesting a lack of sensitivity to changes in emotional expression. Further eye-tracking studies will be needed to explore attention to social stimuli and to better understand deficits in young children with autism, given the clinical heterogeneity of this population.

## Data Availability Statement

The raw data supporting the conclusions of this article will be made available by the authors, without undue reservation.

## Ethics Statement

The studies involving human participants were reviewed and approved by the Local Research Committee, the Commission Centrale d'Ethique de Recherche (CCER) in Geneva, Switzerland. Written informed consent to participate in this study was provided by the participants' legal guardian/next of kin.

## Author Contributions

MF, MS, and BG designed the study. MF and NK contributed to the clinical and eye-tracking data collection. AB analyzed the data and wrote the manuscript. MF, NK, BG, and MS contributed to the interpretation of the results and to the writing of the manuscript. All authors have approved the final manuscript.

## Conflict of Interest

The authors declare that the research was conducted in the absence of any commercial or financial relationships that could be construed as a potential conflict of interest.
